# Predictors of evidence-based practice competency among Tunisian nursing students

**DOI:** 10.1186/s12909-022-03487-4

**Published:** 2022-06-02

**Authors:** Mohamed Ayoub Tlili, Wiem Aouicha, Syrine Tarchoune, Jihene Sahli, Mohamed Ben Dhiab, Souad Chelbi, Ali Mtiraoui, Thouraya Ajmi, Mohamed Ben Rejeb, Manel Mallouli

**Affiliations:** 1 University of Sousse, Faculty of Medicine of Sousse, Department of Family and Community Medicine, LR12ES03, 4002 Sousse, Tunisia; 2 University of Sousse, Higher School of Health Sciences and Techniques of Sousse, 4054 Sousse, Tunisia; 3 University of Sousse, Faculty of Medicine of Sousse, 4002 Sousse, Tunisia; 4 Sahloul University Hospital, Department of Prevention and Care Safety, 4054 Sousse, Tunisia

**Keywords:** Evidence-based practice, Nursing education, Nursing knowledge, Nursing practice, Quality of care, Patient safety

## Abstract

**Background:**

Evidence-based practice (EBP) is an important competency of undergraduate nursing students which should be cultivated before graduation by increasing future healthcare providers’ knowledge, skills and attitudes towards EBP. This study aimed to describe nursing students’ competencies (attitudes, knowledge, skills) in Evidence-based practice (EBP) and to determine factors predicting EBP competency.

**Methods:**

A descriptive cross-sectional study was conducted at the Higher School of Health Sciences and Techniques of Sousse (Tunisia) among 365 nursing students. Data were collected using the validated Evidence Based Practice Competencies Questionnaire (EBP-COQ). Multiple linear regression was performed to determine factors predicting EBP competencies.

**Results:**

The overall score of EBP-COQ questionnaire was 3.26 ± 0.53 out of 5. The attitude, skills and knowledge subscales received 4.04 ± 0.41; 3.05 ± 0.77 and 2.70 ± 0.74 as mean scores respectively. Multiple linear regression analysis (table 4) revealed that significant related factors were academic level (β = 0.271, *p* = 0.001), English-language reading skills (β = 0.435, *p* < 0.001), facing staff resistance in implementing a new evidence-based procedure (β = − 0.081, *p* = 0.035) difficulties in obtaining full-text papers (β = − 0.127, *p* < 0.001) and training in methodology (β = 0.232, *p* < 0.001) and also in statistics (β = 0.205, *p* < 0.001).

**Conclusions:**

These results help to understand students’ attitudes, knowledge and skills in EBP and can be therefore a starting point to develop effective strategies for EBP curricula.

**Supplementary Information:**

The online version contains supplementary material available at 10.1186/s12909-022-03487-4.

## Background

During the last decades, Evidence-Based Practice (EBP) has become an indispensable part of healthcare and essential to provide patients with safe care, worldwide [1, 2]. Changes in healthcare services in response to the ever changing public health needs have resulted in a significant increase regarding the importance of an evidence-based care [3]. Furthermore, as nurses are becoming more involved and concerned by clinical-decisions making, it is increasingly necessary for them to make effective and justifiable decisions based on the best evidence [4].

EBP has been proven to enhance patient safety, clinical outcomes, and healthcare costs, and it is regarded as a critical component and valuable tool in improving healthcare quality [5–7].

Moreover, several researchers have emphasized that EBP is not only an important competency of graduated healthcare providers but also should be cultivated before graduation by increasing undergraduate healthcare students’ knowledge, skills and attitudes towards EBP [8–11]. Indeed, in order to improve their likelihood of using EBP in their future practice, students must be well-equipped with EBP competencies when transitioning from studentship to professional health-caring practice, i.e. students need to be equipped with adequate knowledge, skills and attitudes within the clinical setting to effectively conduct research and implement evidence-based changes [10, 12]. In other words, students are in a strategic position to affect the implementation of EBP in the healthcare sector [12]. Hence, integration of EBP in academic curricula was recommended [13]. Indeed, an early introduction was shown to help equip nurse students with favorable attitudes about research and evidence-based care, which contributes to more robust EBP implementation in the future [9]. Eizenberg M reported that nurses whose college program included EBP modules were more likely to use EBP [12].

In the other hand, despite efforts to optimize the uptake of research findings into clinical practice several education providers report that undergraduate nursing students still fail to see the importance of theory and evidence in their everyday activities [8]. In their research, Cruz et al. [14] found that conventional nursing research programs using research textbooks have resulted in a lack of clarification about EBP content, process, and outcomes. And as a result, students leave their courses with little or no motivation to continue reading, evaluating, using, and implementing evidence from research. In low- and middle-income countries (LMICs), the situation is further exacerbated and limited integration of EBP in education and clinical practice is often observed, owing to the longstanding shortage of human or material resources [9]. Actually, in LMICs, the concept of EBP has also been embraced but with significant hurdles to its implementation; information seeking and retrieval abilities of healthcare workers have been reported to be poor, and deficiencies in the use of updated information resources have been noted [15]. This, in turn, can hamper the poor level of quality of care and patient safety that these countries already suffer from. In countries faced with these circumstances, the need to implement effective and evidence-based healthcare strategies becomes even more important [16].

Despite the recognized need for exploring EBP in LMICs that could facilitate interventions and health policy directions aiming at optimizing best practice in these settings, there is a shortage of studies concerning EBP and in particular among nursing students in these countries. Insights into predictors of EBP competencies among nursing students in LMICs are the indeed first step to designing effective interventions for successful implementation of EBP [10, 12].

Furthermore, several studies have shown the relationship between the EBP competencies (knowledge, attitudes and skills). For example, Stokke et al. [2] reported that attitudes towards EBP tend to vary with knowledge and skills. According to Melnyk et al. [17]., as well as to Saunders & Vehviläinen-Julkunen [18], the lack of knowledge of EBP steps and principles can lead to negative attitudes towards EBP and results in their limited application or even no application at all. This lack of knowledge is often associated with limited training in research and EBP [19]. In the same path, Tacia et al. [20] reported that a lack of advanced literature search skills and critical appraisal may lead to negative attitudes towards EBP, which may be minimized by appropriate teaching and practice of these skills [17]. However, the majority of these studies concerned clinical staff and rare are the studies that reported relationship between these three competencies among undergraduate nursing students.

In the Tunisian context, the concept of EBP is not widely known and nursing professionals rarely rely on research to guide the care they provide.. Furthermore, there is a dearth of studies that address the EBP and to the best of our knowledge, no previous study had investigated Tunisian nursing students’ EBP competencies.

## Methods

### Study aim, design, settings and participants

This study aimed to describe nursing students’ EBP competencies (attitudes, knowledge, skills) and to determine the factors associated with EBP competency. It is a descriptive cross-sectional study carried out from February to March 2018 at the Higher School of Health Sciences and Techniques of Sousse (HSHSTS), Tunisia. The target population for this study consisted of the undergraduate nursing students enrolled in first, second and third year of their bachelor degree and also of first and second year of Master of research students.

### Study instrument

The Evidence-Based Practice Competence Questionnaire (EBP-COQ) [21] was used in this study. This questionnaire evaluated the competence in EBP and consists of 25 items, which are organized in three subscales: Attitudes toward EBP (13 items); Skills in EBP (6 items) and Knowledge in EBP (6 items). All items of the instrument are scored on a Likert-type scale ranging from 1 (Strongly disagree) to 5 (Strongly agree) and students were asked to indicate how much they agree or disagree with each item.

This tool showed favorable psychometric properties; Cronbach’s alpha for the entire questionnaire was 0.888, and the value for each factor was 0.940 for attitudes toward EBP, 0.756 for EBP skills, and 0.800 for EBP knowledge [21]. The test–retest reliability values for EBP-COQ subscales were 0.95 for attitudes, 0.93 for skills, and 0.96 for knowledge [21].

Reliability was tested for this sample of Tunisian nursing students by measuring internal consistency. Cronbach’s Alpha was of 0.858 for attitudes subscale, 0.871 for skills subscale and 0.844 for knowledge subscale and 0.9 for the whole questionnaire.

Background and predictive variables were chosen based on previous studies [14, 22–27].

### Data collection and ethical considerations

Prior to data collection, an authorization was signed by the general secretary of the HSHSTS.

The questionnaires were administered in the classroom to each participant accepting to take part in the study after signing an informed consent. This study was conducted with respect to the ethical standards regarding confidentiality and anonymity. Besides, a signed permission from the author of the EBP-COQ was obtained before its use for the purpose of this study. Also, the survey was approved by the ethics committee of the HSHSTS, where the study was conducted (Reference number: ESSTSSo_01/2017).

All methods were carried out in accordance with relevant guidelines and regulations.

### Data analysis

Data entry and analysis were performed using the Statistical Package for Social Sciences (SPSS) version 20. Both descriptive and inferential statistical tools were used to analyze the data collected. Descriptive statistics including frequencies and percentages were used for categorical variables. For quantitative data, means and standard deviations (SD) were used to describe the sample characteristics.

Regarding the EBP competence questionnaire (EBP-COQ), a mean score for each factor as well as for the whole questionnaire was calculated.

Independent-Samples T test, One-way ANOVA test were used to assess the differences in knowledge, attitudes, skills towards EBP and the global EBP score between the different groups in categorical variables. The association between age and the different EBP subscales as well as relationships between the EBP-COQ components was assessed using Pearson correlation. Then, all variables significantly associated with EBP competency in the bivariate tests were included in a multiple linear regression model to identify significant predictors of EBP competency after assessing the normality of EBP-COQ data. All statistical analyses were two tailed and the statistical significance point p was set at 0.05.

## Results

Out of 392 nursing students enrolled at the HSSTHS, a total of 365 students completed the survey of our study with a response rate of 93.1%. The mean age of the participants was 20.93 ± 1.48 years. The overwhelming majority of the participants were females (86%). The majority of respondents (61.6%; *n* = 223) were familiar with the meaning of EBP. Characteristics of participants are represented in Table 1.

**Table 1:** Characteristics of participants

Characteristics		
**Age**	**m (±SD)**	**[Min-Max]**
	20.93 (±1.48)	[18-27]
	**Frequency (n)**	**Percentage (%)**
**Gender**		
Male	51	14
Female	314	86
**Academic degree**		
Bachelor	324	88.8
Master	41	11.2
**Bachlor degree level**		
1st year	105	28.8
2nd year	92	25.2
3rd year	127	34.8
**Master degree level**		
1st year	22	6
2nd year	19	5.2
**English-language reading skills**		
Poor	0	0
Below average	39	10.7
Average	166	45.5
Above average	122	33.4
Excellent	38	10.4
**Training in research methodology**		
Yes	249	68.2
No	116	31.8
**Training in statistics**		
Yes	178	48.8
No	187	51.2
**Familiarity with the term EBP**		
Yes	254	69
No	114	31
**Framework of familiarity with EBP**		
Basic training course in the university	150	67.3
Participation in conferences/seminars on EBP	48	21.5
Self-training (Scientific papers, books...)	25	11.2
**Facing staff resistance in implementing/ adopting a new EBP during internship**		
Yes	242	66.3
No	123	33.7

### The evidence-based practice competence questionnaire (EBP-COQ)

The overall score for the EBP-COQ questionnaire was 3.26 ± 0.53 out of 5, having 1.92 as minimum score and 4.47 as maximum score. The attitude subscale received the highest mean score (4.04 ± 0.41) and the skills subscale showed a mean score of 3.05 ± 0.77. The mean score for knowledge subscale was 2.70 ± 0.74. Mean scores for the different subscales and for each item are presented in supplementary file 1.

### Relationships between the EBP-COQ components

Correlations were found between the three self-perceived EBP-COQ components (all at *p* < 0.001). Results of the association between EBP components are reported in Table 2 .

**Table 2:** Relationships between the EBP-COQ components

EBP components		Attitudes subscale	Skills subscale	Knowledge subscale
**Attitudes**	Pearson correlation (r)	1	.456^**^	.274^**^
	Sig (p)		< 0.001	< 0.001
	n	365	365	365
**Skills**	Pearson correlation (r)		1	.678^**^
	Sig (p)			< 0.001
	n		365	365
**Knowledge**	Pearson correlation (r)			1
	Sig (p)			
	n			365

**Correlation is significant at the 0.01 level (2-tailed)

### Factors associated with nursing students’ competencies towards EBP

Bivariate analysis showed that female students had higher mean score than male students in attitudes subscale (4.06 ± 0.40 and 3.94 ± 0.40 respectively; *p* = 0.047), also higher scores in EBP skills and knowledge (*p* = 0.001). Being trained in research methodology and having completed a course in statistics were significantly related with students’ competencies in EBP (*p* < 0.001and *p* < 0.001, respectively), associated with an improvement in their attitudes, skills and knowledge in EBP (Table 3). Multiple linear regression analysis (Table 4) revealed that significant related factors were academic level (β = 0.271, *p* = 0.001),, English-language reading skills (β = 0.435, *p* < 0.001), facing staff resistance in implementing a new evidence-based procedure (β = − 0.081, *p* = 0.035) difficulties in obtaining full-text papers (β = − 0.127, *p* < 0.001) and training in methodology (β = 0.232, *p* < 0.001) and also in statistics (β = 0.205, *p* < 0.001). Results of sensitivity analysis including age and gender are presented in the supplementary file 2.

**Table 3:** Factors associated with EBP (bivariate analysis)

	Attitudes		Skills		Knowledge		EBP competency	
	r	p	r	p	r	p	r	p
**Age**								
r (pearson)	0.089	0.088	0.431	***p <*** **0.001**	0.606	***p <*** **0.001**	0.515	***p <*** **0.001**
	**m (SD)**	**p**	**m (SD)**	**p**	**m (SD)**	**p**	**m (SD)**	**p**
**Sex**								
Female	4.06 (0.40)	**0.047**	3.10 (0.75)	**0.001**	2.75 (0.76)	**0.001**	3.30 (0.53)	**< 0.001**
Male	3.94 (0.40)		2.73 (0.78)		2.38 (0.52)		3.01 (0.41)	
**Academic degree**								
Bachelor degree	4.02 (0.41)	***p <*** **0.001**	2.96 (0.76)	***p <*** **0.001**	2.58 (0.69)	***p <*** **0.001**	3.19 (0.50)	***p <*** **0.001**
Master degree	4.26 (0.34)		3.74 (0.45)		3.63 (0.43)		3.88 (0.28)	
**Bachelor level**								
First year	3.94 (0.46)	0.733	2.61 (0.80)	***p <*** **0.001**	2.10 (0.46)	***p <*** **0.001**	3.19 (0.42)	***p <*** **0.001**
Second year	3.99 (0.57)		2.92 (0.74)		2.53 (0.59)		3.40 (0.44)	
Third year	4.01 (0.36)		3.35 (0.78)		3.10 (0.62)		3.62 (0.41)	
**Master level**								
First year	4.29 (0.32)	0.553	3.88 (0.42)	**0.037**	3.50 (0.33)	**0.042**	4.01 (0.28)	0.673
Second year	4.22 (0.36)		3.59 (0.43)		3.78 (0.49)		3.97 (0.31)	
**English language skills**								
Below average	3.50 (0.30)	***p <*** **0.001**	2.46 (0.51)	***p <*** **0.001**	2.26 (0.46)	***p <*** **0.001**	2.74 (0.34)	***p <*** **0.001**
Average	4.04 (0.41)		2.90 (0.72)		2.49 (0.56)		3.14 (0.42)	
Above Average	4.16 (0.30)		3.27 (0.77)		2.97 (0.82)		3.47 (0.54)	
Excellent	4.25 (0.28)		3.61 (0.60)		3.14 (0.68)		3.67 (0.47)	
**Training in research methodology**								
Yes	4.08 (0.35)	**0.026**	3.28 (0.63)	***p <*** **0.001**	2.95 (0.69)	***p <*** **0.001**	3.44 (0.47)	***p <*** **0.001**
No	3.97 (0.49)		2.56 (0.80)		2.14 (0.49)		2.89 (0.45)	
**Training in statistics**								
Yes	4.12 (0.35)	**0.001**	3.42 (0.64)	***p <*** **0.001**	3.14 (0.72)	***p <*** **0.001**	3.56 (0.48)	***p <*** **0.001**
No	3.97 (0.44)		2.70 (0.72)		2.28 (0.46)		2.98 (0.40)	
**Familiarity with EBP**								
Yes	3.45 (0.52)	***p <*** **0.001**	4.14 (0.33)	***p <*** **0.001**	3.28 (0.71)	***p <*** **0.001**	2.92 (0.77)	***p <*** **0.001**
No	2.98 (0.42)		3.90 (0.47)		2.70 (0.72)		2.34 (0.51)	
**Facing staff resistance in implementing/ adopting a new EBP during internship**								
Yes	3.93 (0.49)	***p <*** **0.001**	3.00 (0.75)	**0.014**	2.62 (0.72)	***p <*** **0.001**	3.39 (0.46)	***p <*** **0.001**
No	4.15 (0.39)		3.21 (0.80)		2.93 (0.75)		3.63 (0.45)

**Table 4:** A multiple linear regression analysis of evidence-based practice competency

Explanatory factors	B	SE	β	t	***p*** value	95% CI
**Academic level**	.118	.035	.271	3.326	.001	(.048- .187)
**English-language skills**	.258	.025	.435	10.468	< 0.001	(.210- .307)
**Facing staff resistance and difficulty in implementing/ adopting a new evidence-based procedure during internship**	−.083	.039	−.081	−2.117	.035	(−.159- -.006)
**Training or education in research methodology**	.206	.042	.232	4.431	< 0.001	(.154 - .361)
**Education in statistics**	.198	.046	.205	4.323	< 0.001	(.108 - .288)
**Difficulties in obtaining full-text papers or accessibility to scientific publications**	−.176	.053	−.127	−3.300	.001	(−.281- .071)

Dependent variable: EBP-Competency (overall score); R^2^: .523; Adjusted R^2^: .514; F: 55.576; *p* < 0.001

## Discussion

### EBP scores

The results from the EBP-COQ used in our study to assess nursing students’ competencies towards Evidence-Based Practice, revealed that the general outline of the findings indicates a positive attitude toward this approach, as implied by the high score reported in the EBP-COQ attitude subscale (4.04 ± 0.41 out of 5), although there is room for improvement. The findings above comply with what Martinez et al. [23] and Yildiz & Güngörmüş [28] found out while using the same instrument. Being aware of the differences between these three studies and the fact that EBP is a concept that is very recently introduced in Tunisia in comparison with other contexts, it is interesting to underline that the scores obtained in the attitude subscale were thereabouts similar to ours. This could be attributed to the general positive connotation towards the term evidence-based practice itself in the sense that the majority want their self-practices to be based on the best practice recommendations from the latest scientific research results., regardless of the level of their mastery of skills and knowledge relating to this concept (such as PICOTs questions, bibliographic research and critical analysis of articles).

Implementing new knowledge is, however, not a straightforward and easy process, as it involves both organizational and individual factors. Studies from Norwegian physiotherapy [29] and pharmacy students [30], and also among Saudi dental and medical [31] and nursing students [14], have shown that attitudes and beliefs about the benefit of EBP are often rated high, whereas knowledge and skills related to EBP are rated low or moderate.

In this study, the results with regard to the students self-confidence in their EBP skills and knowledge seem insufficient compared to those in Spain [23] and Turkey [28]. This aforementioned difference can be explained by the fact that EBP was already implemented for years as a teaching module in these two countries while in the current study, EBP concept remained foreign and new for the questioned Tunisian nursing students.

### Relationships between the EBP-COQ components

Results show that the attitude domain was correlated with skills and with knowledge domains. This suggests that the more students perceive that they are well equipped with the necessary EBP knowledge and have better skills, the more they have better attitudes towards EBP. This finding offers support to Labrague et al.’s study that reported a positive relationship between the three EBP competencies (knowledge, skills, and attitudes) [32].

These results have implications for nursing education and, as for Labrague et al., may assist and guide nursing faculty in selecting appropriate measures that would best enhance EBP competence among nursing students and also guide transformation for nursing education curricular of faculties [32].

### Factors associated with EBP

The current research indicated that students with greater English-language reading skills reported EBP competency. These findings are consistent with previous studies conducted among Italian [33] and Taiwanese [27] undergraduate healthcare students, suggesting that English reading abilities are indeed a hindrance and create distinct challenges for non-native English speakers to utilize research evidence; since English is generally considered as the universal language of the scientific community [34].

The attendance to a research methodology course and statistics training has emerged to substantially affect students’ competencies towards EBP. Findings by Ashktorab et al. [22] support our results and report that familiarity with statistics and research methods primarily affects student’s knowledge and attitudes towards EBP. Although, these trainings provide students with the basic’s knowledge and necessary skills in understanding and conducting researches. Also, According to Cruz et al. [14], attending trainings/seminars on research and EBP may improve awareness on this concept, thus encouraging positive beliefs towards it. Several other studies have reported an improvement in the EBP beliefs after implementing EBP educational interventions among health practitioners [35–37] and students [23, 38].

Otherwise, inadequate knowledge about research methods, inability in evaluating the quality of research, lack of skills in critical appraisal, unfamiliarity with the research language and inability in understanding statistical analysis served as hindrances in implementing EBP among students [24–26, 29, 39] as for healthcare practitioners [40–42].

Results also showed that difficulties in obtaining full-text papers was significantly associated with a decreased EBP competency. Actually, the problem of accessibility and availability of research data remain a problem especially for scholars from low resource countries and it is a subject that always arises. Accessible knowledge is an important first step in the translation of knowledge and its implementation in practice, especially in LMICs [43]. In fact, research environments in many LMICs differ markedly from high-income countries (HICs) in terms of resource provision, research support and infrastructures which makes access to scientific papers complicated [44]. This difficulty of access to research faced by healthcare workers from LMICs makes the implementation of EBP very difficult. This restriction of access to knowledge emerging from public health research, which can have a direct impact in life or death situations, continues to be a critical social justice issue [43].

Smith et al.’s article extensively discussed the disparity and inequity between researchers from HICs and those from LMICs when knowledge, because of better resources, remains with HIC research groups and is published in expensive subscription journals, held behind ‘paywalls’, and affordable mainly for HIC researchers [43].

Mutual capacity building is needed so that researchers from both HICs and LMICs may learn from one another and the intended outcome is to achieve better global health equity across individuals and nations [43]. One of the solutions that can help reduce this problem of inequity is Open Access (OA) where scientific content is freely available online to all readers leading to wider accessibility and resulting in a better uptake from research to practice [45].

Furthermore, in our study, 66.3% of students find that there is a culture of resistance to change and that they face resistance, unacceptability and difficulty in implementing and adopting a new evidence-based procedure during internship in clinical placement. This resistance was associated with a negative EBP competency. It is important to note that this resistance was reported by the respondents subjectively, based on their personal experiences and general perceptions and impressions. Dubois et al. confirm that in recent years, work reorganization initiatives have been implemented in many healthcare facilities to improve work processes, and increase efficiency, but despite claims regarding the potential benefits of these initiatives for healthcare providers professionals, institutions often encounter difficulties and unforeseen setbacks in their implementation [46]. The failure of many of these change efforts has often been attributed to a culture of resistance to change [46]. This resistance can equip students, during their internship, with a feeling that despite their motivation to change practices according to new evidence-based knowledge, they cannot change and improve practice in the face of resistance from senior staff. Thus, their effort to implement EBP may confront them with problems and conflicts early in their careers. In this lane, Iradukunda & Mayers [9] revealed, that students in their study reported lack of support and example from lecturers, clinical instructors and qualified nurses as a barrier to the application of EBP.

As with any research study, there are limitations that can be addressed in future research and that must be highlighted. First, the instrument measured participants’ self-assessments of their attitudes, knowledge and skills in EBP; this may be associated with a reporting bias. Also, the high score reported by students of attitudes for a concept that is not developed enough in Tunisia and on which they do not have a great knowledge can be attributed to a social bias insofar as students are telling investigators what they think they want to hear.

In addition, the interpretation and generalization of the results have to be cautious and carefully discussed since the study was conducted within only one school of health sciences and findings therefore may not be generalized to all Tunisian nursing students.

## Conclusion

The strategic position of nursing students is influential in the adoption of EBP. It is necessary to understand students’ attitudes, knowledge and skills in EBP to be able to develop effective strategies for EBP curricula. Furthermore, understanding the underlying factors is useful in developing teaching strategies for effective EBP.

The EBP-COQ showed a moderately high attitude score towards EBP, but coupled with a low knowledge and skills scores. This could be associated to various factors including academic class levels, education in research methodology and statistics courses and familiarity with EBP. Thereby, equipping students with life-long skills for EBP implementation and improving their perceptions of the value of evidences in practice may increase their likelihood to utilize EBP in their future practice and provide the cost effective, safe, and highest quality care and best outcomes for patients.

Future researchers are recommended to identify barriers that hinder them to adopt EBP in more depth, using a larger sample. Also, further experimental research is encouraged to examine the effectiveness of an educational intervention on EBP among Tunisian healthcare students.

## Supplementary Information

**Additional file 1:** **Supplementary file 1.** Items subscales’ mean scores.

**Additional file 2:** **Supplementary file 2.** A Multiple Linear Regression Analysis of Evidence-Based Practice Competency including age and gender (sensitivity analysis).

## Data Availability

The datasets used and/or analysed during the current study available from the corresponding author on reasonable request.

## Abbreviations

EBP: Evidence-Based Practice
LMICs: Low- and Middle-Income Countries
HSHSTS: Higher School of Health Sciences and Techniques of Sousse
EBP-COQ: Evidence-based Practice Competence Questionnaire
HICs: High-Income Countries
