# Supplementary material for: Predictors of evidence-based practice competency among Tunisian nursing students
Source: BMC Med Educ. 2022 Jun 2;22:421. doi: 10.1186/s12909-022-03487-4 (PMC9161527; doi:10.1186/s12909-022-03487-4)
Supplement: Supplementary file 1 — Additional file 1: Supplementary file 1. Items subscales’ mean scores. [file 12909_2022_3487_MOESM1_ESM.docx]

**Supplementary file 1 : Items subscales’ mean scores**

| **Items’ subscales** | **Mean (SD)** |
| --- | --- |
| **ATTITUDES SUBSCALE** | **4.04 (.41)** |
| A1) The EBP helps to make decisions in clinical practice. | 4.28 (.63) |
| A2) I’m confident that I will be able to evaluate critically the quality of a scientific article. | 3.56 (.79) |
| A3) The practice of EBP will help to have a better definition of the nurse role. | 4.11 (.68) |
| A4) The nursing contract should include time to read scientific papers and make critical appraisal of them. | 3.61 (.8) |
| A5) The widespread EBP implementation will allow to increase nursing autonomy from others professions. | 4.06 (.74) |
| A6) When I work as a nurse I will please if the PBE will be in practice. | 4.14 (.83) |
| A7) The application of EBP improves patient´s healthcare outcomes. | 4.64 (.7) |
| A8) In the future I wish to contribute to apply the EBP. | 3.83 (.89) |
| A9) I do not like reading scientific articles. | 3.95 (.97) |
| A10) The patient care will experiment minor changes with the EBP application. | 3.72 (.67) |
| A11) It pleased me that the EBP is only a theoretical movement that does not take in practice. | 3.76 (.74) |
| A12) If I will have the opportunity, I would assist to an EBP course. | 3.95 (.74) |
| A13) I would like to have better access to published nursing evidences. | 4.53 (.77) |
| **SKILLS SUBSCALE** | **3.05 (.77)** |
| S1) I feel able to make a clinical question to start the searching of the best scientific evidence. | 3.42 (.9) |
| S2) I do not feel able to search for scientific evidences in a structured and systematic way in the main health sciences databases (MedLine, PsycINFO, CINALH..) | 3.47 (1.03) |
| S3) I do not feel able to search for the scientific evidences in the most important systematic reviews and clinical practice guidelines databases. | 3.39 (1.14) |
| S4) I feel able to evaluate critically the quality of a scientific article. | 3.01 (.98) |
| S5) I do not feel able to analyze if the obtained results of a scientific study are valid. | 2.59 (1) |
| S6) I feel able to analyze the practical utility of a scientific study. | 2.54 (.88) |
| **KNOWLEDGE SUBSCALE** | **2.7 (.74)** |
| K1) I know how to make clinical questions organize in the PICO format (Patient/population, Intervention/indicator, Comparison/control and Outcome). | 2.31 (1) |
| K2) I know the principal sources that offer the information revised and catalogued behind the evidence point of view (Joanna Briggs Institute, Cochrane Library, Evidence Based-Nursing, etc. | 3.56 (1.04) |
| K3) I do not know the most important characteristics of the principal study designs in research. | 2.59 (1.02) |
| K4) I know the different evidence level of the study designs in research | 3.07 (1) |
| K5) I do not know the different recommendation grades about the adoption of a determined procedure or health intervention. | 2.4 (.88) |
| K6) I know the principal measures of association (RR, OR, etc…) and potential impact (NNT, NNH, ARR, etc.) that allow to evaluate the magnitude of the analyzed effect in studies. | 2.44 (1.03) |
