# Supplementary material for: Predictors of evidence-based practice competency among Tunisian nursing students
Source: BMC Med Educ. 2022 Jun 2;22:421. doi: 10.1186/s12909-022-03487-4 (PMC9161527; doi:10.1186/s12909-022-03487-4)
Supplement: Supplementary file 2 — Additional file 2: Supplementary file 2. A Multiple Linear Regression Analysis of Evidence-Based Practice Competency including age and gender (sensitivity analysis). [file 12909_2022_3487_MOESM2_ESM.docx]

**Supplementary file 2 : A Multiple Linear Regression Analysis of Evidence-Based Practice Competency including age and gender (sensitivity analysis)**

| **Explanatory factors** | **B** | **SE** | **β** | **t** | **p value** | **95% CI** |
| --- | --- | --- | --- | --- | --- | --- |
| **Age** | .148 | .057 | .111 | 2.586 | .010 | (.260 ; .035) |
| **Gender** | .036 | .018 | .083 | 2.041 | .042 | (.071 ; .001) |
| **Academic level** | .111 | .040 | .255 | 2.741 | .006 | (.031 ; .190) |
| **English-language skills** | .252 | .024 | .424 | 10.643 | <0.001 | (.205 ; .298) |
| **Facing staff resistance and difficulty in implementing/ adopting a new evidence-based procedure during internship** | -.081 | .039 | -.079 | -2.065 | .040 | (-.157 ; -.004) |
| **Training or education in research methodology** | .116 | .055 | .113 | 2.099 | .036 | (.007 ; .225) |
| **Education in statistics** | .199 | .046 | .206 | 4.351 | .000 | (.109 ; .289) |
| **Difficulties in obtaining full-text papers or accessibility to scientific publications** | -.173 | .054 | -.125 | -3.231 | .001 | (-.279 ;-.068) |

| Dependent variable: EBP-Competency (overall score); R^2^ : .524; Adjusted R^2^ : .514; F: 48.943 ; p<0.001 |
| --- |
